# Probiotics and magnesium orotate for the treatment of major depressive disorder: a randomised double blind controlled trial

**DOI:** 10.1038/s41598-024-71093-z

**Published:** 2024-09-06

**Authors:** Esben Strodl, Matthew Bambling, Sophie Parnam, Gabrielle Ritchie, Susanna Cramb, Luis Vitetta

**Affiliations:** 1 School of Psychology and Counselling, Queensland University of Technology, Brisbane, Australia; 2 Faculty of Medicine, University of Queensland, Brisbane, Australia; 3 Australian Centre for Health Services Innovation, School of Public Health and Social Work, Queensland University of Technology, Brisbane, Australia; 4 Faculty of Medicine and Health, University of Sydney, Sydney, Australia

**Keywords:** Clinical microbiology, Psychology

## Abstract

Following on from our pilot studies, this study aimed to test the efficacy of a combination of probiotics (*Lactobacillus acidophilus*, *Bifidobacterium bifidum*, *Streptococcus thermophilus*), magnesium orotate and coenzyme 10 for the treatment of major depressive disorder (MDD) through a double-blind placebo controlled clinical trial. The participants were 120 adults diagnosed with MDD randomised to daily oral administration, over 8 weeks, of either the intervention or placebo, with a 16-week follow-up period. Intent-to-treat analysis found a significantly lower frequency of the presence of a major depressive episode in the intervention group compared with placebo at the end of the 8-week treatment phase, with no difference between the two conditions at 8-week follow-up. Both the categorical and continuous measure of depressive symptoms showed a significant difference between the two conditions at 4 weeks, but not 8 and 16 weeks. The secondary end-point was demonstrated with an overall reduction in self-rated symptoms of anxiety and stress in the active treatment group compared with placebo. These findings suggest that the combination of probiotics, magnesium orotate and coenzyme 10 may be an effective treatment of MDD over an 8-week period.

Globally, major depressive disorder (MDD) is one of the leading causes of disability^1^. Lifetime prevalence rates range between 10%^2^ and 20%^3^, with incidence rates surging worldwide during COVID-19^4^. While treatment guidelines recommend antidepressant medication and psychotherapy standardised interventions for MDD^5,6^, these interventions have consistently shown only a small beneficial treatment effect compared with placebo^7^. Specifically, 37% of MDD patients who receive first-line pharmacological treatment do not achieve a response within 6 to 12 weeks, and 53% do not achieve remission^8^. Similarly, while 62% of MDD patients treated with psychotherapy no longer meet criteria for MDD after treatment, when compared with control conditions, psychotherapy only adds advantage to 19% of MDD patients^9^. In addition, adverse events can be high. For example, greater than a third of individuals taking selective serotonin reuptake inhibitors for depression report adverse events^10^. Such findings indicate the need to explore alternative treatment options for MDD.

One promising alternative treatment for depression is the use of probiotics. However, while there is meta-analytic evidence to support the use of probiotics to reduce depressive symptoms in subclinical populations^11^, there have only been a small number of randomised clinical trials to determine the utility of probiotics for the treatment of MDD resulting in meta-analyses with mixed results^12,13^. Similarly, a recent meta-analysis examined 13 studies found that probiotics have a positive effect on depression, with a stronger effect size in clinical samples than community samples, although the authors noted the dearth of published studies using clinical samples^14^. Another recent meta-analysis of randomized controlled trials demonstrated while many trials show positive outcomes there is considerable variability in the results and highlighted the need for more empirical research^15^. To date, most probiotic interventions for depression have included strains of the *Bifidobacterium* and *Lactobacillus* species of bacteria^15,16^. However, given the mixed findings for the treatment of MDD, there is a need for further exploration of effective probiotic formula for this condition. Our team has previously found promising results for the addition of magnesium orotate to a probiotic intervention for the treatment of MDD^17^. It has been proposed that magnesium orotate (Mg Orotate) may enhance the effectiveness of probiotic interventions by both a direct influence on intestinal peristaltic and metabolic functions as well as by modulating the gut microbiota^18^. As such the aim of this study was to extend our team’s previous published pilot work by investigating the efficacy of a combined therapy using magnesium orotate and probiotics (*Lactobacillus acidophilus, Bifidobacterium bifidum, Streptococcus thermophilus*) in the management of MDD by means of a double blind RCT comparing NRGBiotic™ with a placebo. Coenzyme Q10 (CoQ10) was included in the NRGBiotic™ formulation given that previous studies suggest that low levels of the coenzyme may have a role in the pathophysiology of depression and in particular in treatment resistant depression and chronic fatigue syndrome that can accompany depression^19,20^. Our hypotheses for this study are as follows.

## *Hypothesis 1:*

The administration of Mg Orotate, CoQ10 and probiotics, over an 8-week period, will result in a greater reduction in the primary outcome measures (frequency of diagnosis of MDD and levels of self-rated depressive symptoms) compared with placebo at 8 weeks. In addition, we anticipate that these differences will be maintained at the subsequent 8-week follow-up period.

## *Hypothesis 2:*

The administration of Mg Orotate, CoQ10 and probiotics, over an 8-week period, will result in greater changes in the secondary outcome measures (reduction in self-rated levels of anxiety and stress) compared with placebo at 8 weeks, and that these differences will be maintained at the subsequent 8-week follow-up period.

## Methods

### Study design

This was a 16-week double-blinded, randomized placebo-controlled clinical trial with two conditions (treatment and placebo). The trial was approved by the Human Research Ethics Committee from the University of Queensland (UQ) (2017000186) and Queensland University of Technology (QUT) (1700001111) and was registered with the Australian and New Zealand Clinical Trials (ACTRN12617000419369) on the 23^rd^ March 2017. All methods were conducted in accordance with relevant guidelines and regulations.

### Participants

Participants were over 18 years old with no upper limit, and a current diagnosis of MDD. The exclusion criteria included: (1) a diagnosis of schizophrenia, bipolar disorder, or current substance misuse disorder, (2) current high suicide risk, (3) current or recent use over the previous 4 weeks of antibiotics, (4) current use of Warfarin, (5) current pregnancy or planning pregnancy over 16 weeks, (6) serious physical illness (e.g., serious life threatening illness or palliative care), (7) current use of antidepressant medication other than SSRIs, or, SNRIs, 8) current or recent use over the previous 4 weeks of probiotics, (9) established or suspected obstructive/central sleep apnoea. Potential participants were also excluded if they were currently taking herbal remedies or medication on the exclusion list (see Supplementary Material A). The significant life events experienced by the participants are reported in Supplementary Material B.

## Measures

### Major depressive disorder

The presence of a MDD was assessed by a clinical psychologist using the research version of the Structured Clinical Interview (SCID-5-RV) for the Diagnostic and Statistical Manual of Mental Disorders (DSM-5)^7,21^. The SCID’s diagnostic sensitivity and specificity for current MDD has been shown to be very good with good inter-rater reliability^22^.

### Depressive symptoms

The severity of depressive symptoms was assessed using the Beck Depression Inventory—II (BDI-II)^23^. The BDI-II has been shown to have good validity and reliability^24^. The internal consistency was very good in this study (α = 0.88).

### Anxiety symptoms

The severity of anxiety symptoms was assessed using the Generalized Anxiety Disorder Scale (GAD-7)^25^. The scale has been shown to have good criterion, construct, factorial, and procedural validity, as well as good internal consistency and test–retest reliability^24^. The internal consistency was good in this study (α = 0.80).

### Stress symptoms

Self-report levels of stress were measured using the 10-item Perceived Stress Scale (PSS)^26^. The PSS has good factorial and criterion validity, good internal consistency, and adequate test–retest reliability^26^. The internal consistency was adequate in this study (α = 0.71).

### Screening for personality disorders

Participants were also asked to complete the Standardized Assessment of Personality (SAPAS)—Abbreviated Scale. The SAPAS is an 8-item dichotomised (Yes/No) self-report screening measure. A score of 3/8 or higher is suggestive of a DSM-5 personality disorder^27^.

### Participant dosage log

Adherence to the study protocol was recorded via documenting daily dosage. Any missed doses, changes to prescribed medication, or notable side effects were also recorded by the research team.

## Procedure

Written informed consent was obtained from all participants prior to enrolment into the trial. Participants completed three stages of screening. The first two stages were completed online and via a telephone interview to determine eligibility. At the final stage, a clinical psychologist administered the research version of the Structured Clinical Interview (SCID-5-RV) to confirm diagnosis of a MDD and obtain a diagnostic profile of current, past, and comorbid psychiatric disorders.

Of the 1610 participants initially assessed for eligibility, 120 participants met the criteria for the study and were randomly allocated to a treatment or placebo condition. The randomisation sequence was generated and stratified by gender (male, female, other) and medication class (no antidepressant, SSRI, SNRI) with a 1:1 allocation using random block sizes of 4 and 6. Participants who met criteria for the study had their details entered into Goji, a web-based trial management system developed at Queensland University of Technology, which utilised a fully automated process for randomisation. All research staff and participants were blinded to group allocation until data collection was completed. The active treatment and placebo capsules were stored separately in amber PET jars with white tamper-evident lids and labelled ‘A’ or ‘B’. Data was collected from the participants at the Queensland University of Technology’s Kelvin Grove Campus in Brisbane Australia. Data collection began July 2018 and ended in May 2020 due to a combination of meeting the minimum sample size required and COVID restrictions and lockdowns making further data collection unfeasible.

**Fig. 1:**
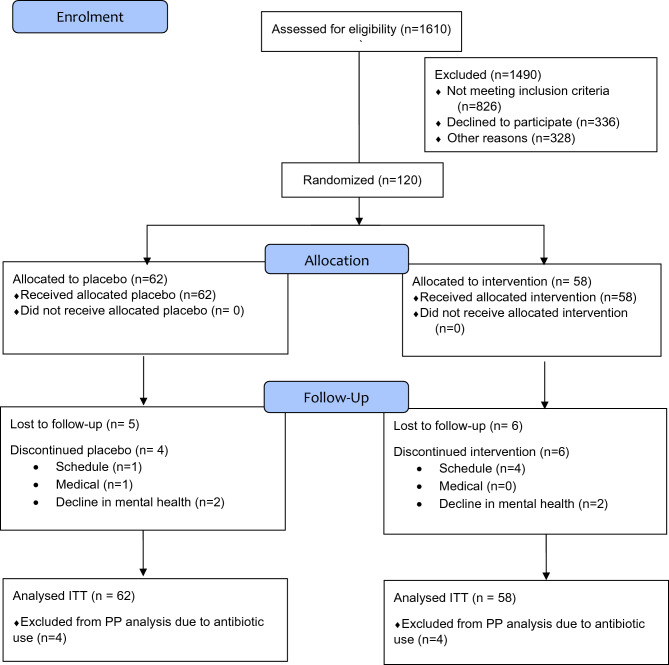
CONSORT Flow Diagram.

Participants were provided with 112 capsules divided evenly across two bottles and a paper dosage log to track adherence and report changes to physical or mental health symptoms, lifestyle, or medication. Participants were instructed to store the bottles in the fridge, administer four capsules daily (two morning and two evening doses with food) and begin administration on the evening of the baseline assessment. At the end of week 4 participants returned the study bottles and remaining capsules were counted and recorded. Participants were given two new bottles of product containing in total 112 capsules of the same condition and returned the remaining capsules at the end of week 8. Between weeks 8 and 16 the intervention ceased, and participants were advised to adhere to the inclusion and exclusion criteria for the duration of this washout period. See Fig. 1 for Consort Flow Diagram. Financial incentives were provided at the screening assessment ($10) and baseline, week 4 and 8 assessments ($20 each). On completion of the week 16 assessment participants were posted one bottle of NRGbiotic™ and a $25 store voucher.

### Study intervention and placebo

NRGBiotic™ is a combination of probiotics with Mg Orotate and CoQ10 (developed and supplied by Medlab Pty Ltd., Botany, NSW, Australia) and was the study intervention. Capsules contained a combination of lyophilized probiotics (*Lactobacillus acidophilus, Bifidobacterium bifidum, Streptococcus thermophilus*) total CFU of 2 × 10^10^, Mg Orotate 1600 mg and CoQ10 150 mg. A pharmacist external to the research team developed the placebo in identical capsules to the NRGbiotic™. These capsules were matched for colour, size, taste, and smell, and consisted of lake carmoisine (red), exalake quinolone (yellow) and rice flour.

## Statistical analysis plan

Baseline differences between the two groups on demographics and descriptive variables were checked using t-tests for continuous variables and chi-square tests for categorical data. Chi-square analyses were run to compare the two groups on the number of participants meeting criteria for MDD post-intervention. Participants who no longer met the criteria for MDD were combined with those who had some subclinical features of MDD but did not meet all the criteria. In addition, chi-square analyses were used to compare the two groups on whether or not they reported at least mild symptoms of depression (i.e., BDI-II > 13). These analyses were conducted using SPSS v26.

The sample size calculation was based on Beck Depression Inventory pre and post scores from our pilot data using the same intervention^17^. The within-subject pooled standard deviation of the difference in responses from baseline to post-intervention was 21 units, and that alpha = 0.05. To identify a clinically important between-treatment difference of 5 units or greater on the BDI-II, with 80% power, we need to obtain complete outcome data on 115 individual participants. Assuming a notional dropout rate or difficulties in collecting later interval measurement data the recruitment target was expanded to at least n = 120 participants.Differences between the two groups in changes over time in the continuous measures of depression, anxiety, stress, and general wellbeing were tested separately using linear mixed models adjusted for baseline values of the relevant dependent variable. Models also included the group assigned (intervention vs placebo) and each follow-up time period (at week 4, 8 and 16) to allow for non-linear patterns. Two model forms were run: one with interactions between the group assigned and follow-up weeks, and one without interactions. Adjusting for antibiotic usage in the 12 months prior to the trial commencing was shown to not improve the model, so was not included. Robust standard errors were used to overcome any misspecification of residual structures.

These analyses were conducted with both intent-to-treat (ITT) and per-protocol (PP) analyses using Stata v16.0 (StataCorp, USA). The ITT analysis for the categorical data involved the last observation carried forward, whereas for the linear mixed models the 5 participants with no outcome data (i.e. no measurements at weeks 4, 8 and 16) could not be included. The PP analyses involved the removal of 8 participants (4 in the intervention group and 4 in the placebo group) who took antibiotics during the 8 week intervention period.

## Results

### Baseline characteristics

The demographic and clinical characteristics of the sample as well as scores on the mental health questionnaires at baseline are reported in Tables 1 and 2. Compared with the participants in the placebo group, the participants in the intervention group were significantly older, experienced later age of onset of first major depressive episode (MDE), drank more caffeinated drinks per day, and had experienced a smaller number of previous psychological conditions. There were no other significant differences between groups for these variables.

**Table 1:** Comparisons between conditions on continuous baseline variables, shown as mean(standard deviation).

Continuous demographic	Placebo *n* = 62	Intervention *n* = 58
Age	35.76 (12.95)	40.58 (13.39)*
BMI	28.13 (6.90)	29.01 (6.82)
Number of comorbid diagnoses	1.58 (1.10)	1.47 (1.21)
Number of past psych conditions	0.89 (0.94)	0.47 (0.66)**
Number of past suicide attempts	0.26 (0.87)	0.27 (0.57)
Number of subthreshold psych conditions	0.24 (0.56)	0.29 (0.59)
Age onset of first MDE	17.60 (10.57)	21.88 (12.96)*
Number of sig negative life events	3.39 (1.45)	3.84 (1.71)
Number of current stressors	1.82 (1.00)	1.64 (1.06)
Physical activity METS (in minutes)	2455.15 (2558.04)	2292.38 (2477.69)
Minute spending sitting during week	373.75 (185.08)	435.54 (228.04)
Global Sleep Score (PSQI)	10.08 (3.17)	10.00 (3.56)
Number of dysbiosis symptoms endorsed	4.23 (2.01)	4.24 (2.11)
Personality Disorder Score (SAPA)	4.06 (1.69)	4.16(1.67)
Standard drinks per day	0.62 (1.00)	0.72 (1.31)
Cigarettes per day	0.75 (3.59)	0.53 (2.36)
Caffeinated drinks per day	1.73 (1.25)	2.05 (1.54)**
Antidepressant dose (mg)	81.00 (67.27)	79.60 (15.41)
Number of weeks on current antidepressant dose	81.91(108.26)	183.14 (323.28)
Number of antibiotic prescriptions in last 12 months	0.71 (.837)	0.72 (1.06)
Depression (BDI-II)	29.81 (10.35)	30.78 (9.55)
Anxiety (GAD-7)	10.95 (4.12)	11.84 (4.96)
Stress (PSS)	25.85 (4.81)	27.12 (4.84)
Diet Energy (kilojoules)	10,688.29 (8121.24)	36,715.85 (212,707.34)
Diet Protein (grams)	101.92 (37.92)	163.96 (559.99)
Diet Total Fat (grams)	98.43 (55.18)	89.34 (28.93)
Diet Carbohydrates (grams)	232.76 (109.90)	641.85 (3296.20)
Diet Total Fibre (grams)	23.19 (11.42)	22.76 (12.17)

Groups compared using t-tests.

*p ≤ .05, **p ≤ .01.

**Table 2:** Comparisons between conditions on baseline categorical demographics.

Categorical demographic		Placebo *n* = 62	Intervention *n* = 58
Gender	Male	20	17
	Female	41	40
	Other	1	1
Relationship status	Married	16	24
	De facto	10	5
	Divorced	6	6
	Dating	12	5
	Single	17	18
	Other	1	0
Current antidepressant	None	37	33
	SSRI	15	17
	SNRI	10	8
Antibiotics in past 12 months	Yes	32	26
	No	30	32
Appendectomy	Yes	4	9
	No	58	49
Intestinal dysbiosis	Yes	52	45
	No	10	13
Previous suicide attempt	No	49	39
	Yes	9	10
Current use of analgesics	Yes	12	13
	No	50	45
Allergies	Yes	33	26
	No	29	32
Physical Activity	Inactive	17	13
	Minimal	26	26
	Health enhancing	19	17
Poor Sleep Quality	Yes	55	52
	No	1	4
History of nicotine use	No	50	46
	Yes	12	12
History of drug use	None	50	44
	Cannabis	7	8
	MDMA	0	1
	More than one	5	4

Groups compared using chi-square tests; no significant differences were found.

### Randomization

In addition to the minimal baseline differences between the two groups, successful randomisation was demonstrated by there being no difference between the two groups at week 4 on their belief as to whether they were in the probiotic or placebo group (χ^2^_(2)_ = 4.76, *p* = 0.12). Similarly, the groups were equivalent in the frequency of participants experiencing a significant medical or life event during the trial (χ^2^_(1)_ = 0.24, *p* = 0.63).

### Adherence

There was no significant difference between the two groups on the number of capsules consumed during the 8 week trial (t_(1, 103)_ = -0.49, *p* = 0.62 [placebo mean (*SD*) = 201.71 (23.79), probiotic mean (*SD*) = 204.48 (32.87)]. There were no significant differences between the two groups on any of the other adherence measures listed in Table 3.

**Table 3:** Comparisons between conditions on adherence variables during trial.

Adherence variables		Placebo *n* = 62	Intervention *n* = 58
Did participant commence, modify, or cease dose of antidepressant during trial?	No	61	56
	Yes	1	2
Medication (other than antidepressant) change during trial	No	31	24
	Yes	31	34
Did participant withdraw prior to completing trial?	No	53	46
	Weeks 1–4	3	3
	Weeks 5–8	3	4
	Weeks 9–16	3	5
	No	55	53

Groups compared using chi-square tests; no significant differences were found.

### Adverse events

No serious adverse events were reported after randomisation. Adverse events (AEs) were reported by participants daily to the clinical staff and were rated in terms of grade, attribution, and outcome and are presented in Supplementary Material C. The placebo group reported 13 adverse events while the intervention group reported 9 adverse events. Some patients experienced multiple AEs. Of the 22 AEs reported, 82% were mild, 18% were moderate and 0% were severe. All AEs were deemed to be possibly attributed to participation in the study. Fifty percent of the AEs were reported to be resolved during the study.

### Changes in major depressive episode diagnosis

All participants met criteria for a current MDE at baseline as an inclusion-criteria for the study. At weeks 8 and 16, the participants were diagnosed as either meeting criteria for a current MDE, or not meeting criteria for MDE using the SCID-5-RV (see Table 4). Using ITT analysis, at week 8 the intervention group had a significantly lower rate of meeting the criteria for a current MDE than the placebo group (χ^2^_(df=1)_ = 3.91, *p* = 0.048) but there was no significant difference between the two groups at week 16 (χ^2^_(df=1)_ = 0.70, *p* = 0.40). However, using the PP analysis, there was no significant difference between the groups in terms of diagnosis at week 8 (χ^2^_(df=1)_ = 4.43, *p* = 0.11) nor at week 16 (χ^2^_(df=1)_ = 1.88, *p* = 0.39).

**Table 4:** Comparisons between conditions on MDE criteria at week 8 and 16.

Met criteria for MDE		Placebo	Intervention
Week 8	Yes	43 (2.0)	30 (− 2.0)
	No	19 (− 2.0)	28 (2.0)
Week 16	Yes	47 (0.8)	40 (− 0.8)
	No	15 (− 0.8)	18 (0.8)

Note: Placebo *n* = 62, Intervention *n* = 58. Adjusted standardised residuals shown in brackets.

### Changes in depressive symptoms

Changes in both categorical and continuous measures of depressive symptoms were examined. When the presence of symptoms of depression were dichotomised into either minimal symptoms (BDI-II <  = 13) or at least mild symptoms of depression (BDI-II > 13), then the intervention group had fewer participants reporting the presence of depressive symptoms at week 4 (χ^2^_(df=1)_ = 4.40, *p* = 0.036). However, there were no significant differences between the two groups at week 8 (χ^2^_(df=1)_ = 2.42, *p* = 0.120) or at week 16 (χ^2^_(df=1)_ = 0.91, *p* = 0.763). See Table 5 for frequencies. The results did not change when using the PP analysis at week 4 (χ^2^_(df=1)_ = 3.91, *p* = 0.048), week 8 (χ^2^_(df=1)_ = 1.57, *p* = 0.210) or week 16 (χ^2^_(df=1)_ = 0.77, *p* = 0.781).

**Table 5:** Comparisons between conditions on presence of depressive symptoms (BDI-II > 13).

	Presence of depressive Symptoms	Placebo	Intervention
Week 4	Yes	43 (2.0)	30 (− 2.0)
	No	19 (− 2.0)	28 (2.0)
Week 8	Yes	37 (1.3)	28 (− 1.3)
	No	25 (− 1.3)	30 (1.3)
Week 16	Yes	40 (0.3)	36 (− 0.3)
	No	22 (− 0.3)	22 (0.3)

Placebo group *n* = 62 Intervention group *n* = 58. Adjusted standardised residuals shown in brackets.

**Fig. 2:**
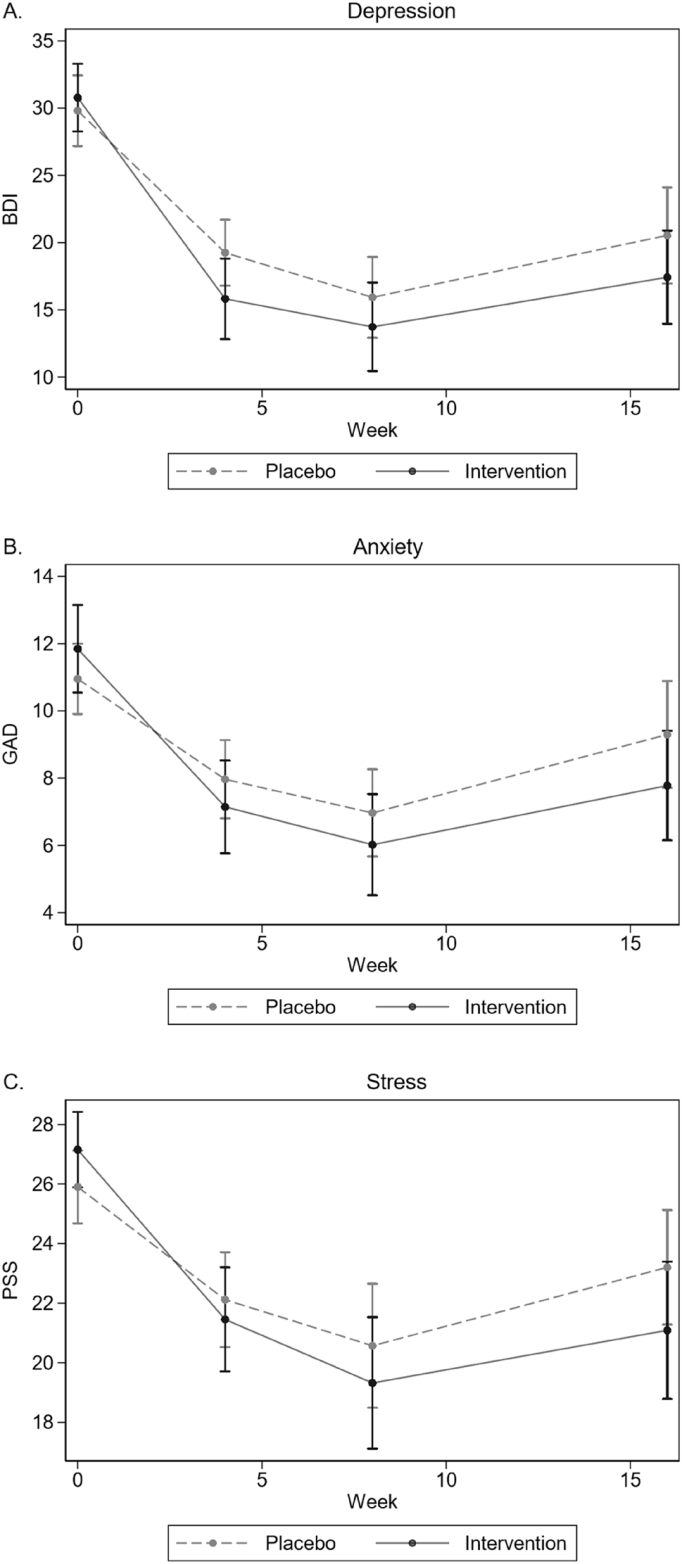
Changes in unadjusted depression scores (BDI-II) over time per group (Placebo n = 62; Intervention n = 58).

A similar pattern of results emerged when using the continuous measure of the BDI-II. Including all participants with at least a baseline measurement (n = 120), depressive symptoms reduced in both the placebo and intervention group within the first eight weeks of the trial, and the intervention group had consistently lower symptoms at all follow-up weeks (Fig. 2a). Nonetheless, using ITT modelled analysis (n = 115), the overall intervention effect of 3.3 BDI points below placebo on average over time was non-significant (Table 6, Fig. 2). At week 4 the intervention group was a significant 3.8 BDI points lower than the control group, but by week 8 this difference had reduced to 2.3 below and was non-significant. At week 16 this difference remained a non-significant 3.5 points lower (Table 6). The per protocol (PP) analysis (n = 108) was conducted by repeating the analysis after removing the eight participants who consumed antibiotics during the 8-week trial (note one of these participants also had no outcome measurements). Results were consistent in the PP analysis, except the overall effect was a significant 3.7 lower on average over time among the intervention group.

**Table 6:** Modelled intervention versus placebo.

d	ITT analysis (N = 115)		PP analysis (N = 108)	
	Coef. [95% CI]	p-value	Coef. [95% CI]	p-value
BDI				
Overall	− 3.26 [− 6.56, 0.04]	0.053	− 3.68 [− 7.11, − 0.24]	0.036
Week 4	− 3.82 [− 7.41, − 0.24]	0.037	− 4.07 [− 7.79, − 0.35]	0.032
Week 8	− 2.35 [− 6.38, 1.69]	0.255	− 2.62 [− 6.79, 1.55]	0.218
Week 16	− 3.53 [− 7.92, 0.85]	0.114	− 4.36 [− 8.85, 0.14]	0.057
GAD				
Overall	− 1.41 [− 2.78, − 0.04]	0.044	− 1.63 [− 3.06, − 0.21]	0.025
Week 4	− 1.30 [− 2.86, 0.25]	0.101	− 1.54 [− 3.09, 0.02]	0.053
Week 8	− 1.20 [− 2.93, 0.52]	0.171	− 1.29 [− 3.04, 0.47]	0.151
Week 16	− 1.76 [− 3.63, 0.12]	0.066	− 2.04 [− 3.97, − 0.11]	0.039
PSS				
Overall	− 2.18 [− 4.11, − 0.24]	0.027	− 2.08 [− 4.06, − 0.10]	0.040
Week 4	− 1.72 [− 3.86, 0.41]	0.113	− 1.56 [− 3.73, 0.62]	0.160
Week 8	− 2.05 [− 4.64, 0.54]	0.121	− 1.71 [− 4.33, 0.91]	0.201
Week 16	− 2.89 [− 5.42, − 0.37]	0.025	− 3.12 [− 5.73, − 0.52]	0.019

*BDI* Beck Depression Inventory II, *GAD* Generalized Anxiety Disorder Scale, *PSS* Perceived Stress Scale, *Coef.* coefficient and represents the modelled average difference between placebo and intervention groups.

Overall estimates obtained from models adjusted for baseline score and follow-up weeks. By follow-up week estimates obtained from models further including an interaction between intervention/placebo groups and follow-up weeks.

### Changes in anxiety symptoms

Anxiety symptoms reduced during the first 8 weeks of the trial for both the placebo and intervention group, and the intervention group had consistently lower symptoms at all follow-up weeks (Fig. 2b). While overall intervention effects on average over time were significantly lower (− 1.4 GAD-7 points) compared to placebo, under ITT there were no significant differences observed at week 4, 8 or 16 (Table 6). This differed from the per-protocol analyses which had a significantly lower GAD-7 score among the intervention group at week 16.

### Changes in stress symptoms

Again, both the placebo and intervention group showed decreases in stress symptoms within the first eight weeks of the trial, with consistently lower symptoms at all follow-up weeks in the intervention group (Fig. 2c). Under both ITT (-2.2 PSS points) and per protocol analysis (-2.1 PSS), overall effects on average over time were significantly lower among the intervention group compared to placebo (Table 6). The only specific week with significantly lower estimates for the intervention group was in week 16.

## Discussion

This study aimed to test the hypotheses that an eight-week intervention involving the daily administration of probiotics (*Lactobacillus acidophilus, Bifidobacterium bifidum*, *Streptococcus thermophilus*), magnesium orotate and coenzyme Q10, would result in a significant reduction in the diagnosis of MDD and depressive symptoms, as well as in the secondary outcomes of symptoms of anxiety and stress, with gains being maintained over a subsequent 8-week follow-up period. The results of this study were mixed.

Using ITT analysis, the intervention group demonstrated a significant reduction in frequency of participants maintaining a diagnosis of MDD at 8 weeks compared to the placebo group. However, this difference was not maintained at the week 16 follow-up period indicating that the effect of the intervention was not sustained. The PP analysis found no significant difference between the two conditions in terms of frequency of diagnosis of MDD at 8 or 16 weeks—possibly due to the reduced sample size reducing the power of the analyses. Interestingly, both the categorical measure of the presence or absence of depressive symptoms, and the continuous measure of depressive symptoms (using the BDI-II), found a significant difference between the two conditions at week 4 but not at weeks 8 and 16, indicating that the strongest effect of the intervention may have occurred at week 4.

When considering the secondary outcome measures, both the ITT and PP analyses found an overall significant reduction in anxiety and stress symptoms in the intervention group compared with the placebo group across time. However, the strength of this effect was not strong enough to identify statistically significant differences between the two conditions at weeks 4, 8 and 16; with the exception of the intervention group reporting lower stress at week 16 (using both ITT and PP analyses) and lower anxiety at week 16 (using PP analyses) than the placebo group.

While our results are consistent with the majority of studies examining the effectiveness of probiotics upon depression and anxiety, a recent systematic review found that a third of studies included failed to find an effect which the authors attributed to the heterogeneity of strains included in the interventions^28^. It is of note therefore that the results of this study are consistent with a similar but smaller RCT by Nikolova et al., which examined effects of a probiotic supplementation adjunctive to antidepressant medication for participants with MDD^29^. This study is particularly interesting to compare results with given that it utilized a similar methodology, and while their intervention involved a wider range of strains of bacteria, it included the three strains of bacteria used in our intervention. Moreover, patients with depression appear to differentiate with healthy controls on the abundance of Streptococcaceae and Bifidobacteriaceae, while there is emerging evidence from intervention trials that changes in depressive symptoms may be associated with increase in the relative abundance of Bifidobacterium^30^.

While we postulate that the key active components of the intervention were the probiotics (*Lactobacillus acidophilus, Bifidobacterium bifidum*, *Streptococcus thermophilus*)*,* it is important to acknowledge that the Mg Orotate and CoQ10 may also have contributed to the effect identified in this study. We have previously demonstrated in an exploratory pilot study that magnesium orotate may have antidepressive actions^31^. Furthermore, in a second pilot study we demonstrated that the combination of Mg Orotate plus CoQ10 plus probiotics also showed efficacy in reducing major depression symptoms^17^. Therefore, given the results observed in our previous pilot studies we do acknowledge that the combination of compounds could have an add-on effect in improving major depression symptoms. We also note that other smaller studies have found an effect for probiotics without the inclusion of Mg Orotate and CoQ10^29,30^.

To-date, our study is the largest double-blind RCT to examine the efficacy of probiotics for the treatment of MDD. Given the small number of RCTs into probiotics and MDD and the mixed findings from current meta-analyses, the results of our study strengthen the literature that probiotic interventions can produce short term improvements in symptoms of depression, anxiety, and stress in adults with MDD. Moreover, our results indicate that the intervention group did not experience any significant AEs compared with the control group. However, given the effects upon depressive symptoms were not maintained at the week16 follow-up, our results also suggest a few possible reasons for the lack of maintenance that need to be investigated in future trials. First, it is possible that a dosage of 8 weeks is not long enough to support long term colonisation. Second, it is possible that colonisation may be improved by symbiotic intervention of both prebiotics and probiotics^32^. Third, it is possible that treatment with the probiotics may only be needed for the first 8 weeks, but that a longer dosage of magnesium orotate is needed to enhance its proposed effects upon the effectiveness of probiotis^33^. Fourth, given that adults with MDD with and without high levels of anxiety demonstrate differences in relative abundance of particular species of gut microbiota^34^, it is possible that the intervention had a differential impact upon the MDD participants with and without high anxiety.

While our study demonstrated significant reductions in depressive, anxiety and stress symptoms in our intervention group compared with placebo, there are limitations that affect the interpretation of the findings. First, the placebo group also experienced significant improvements on our mental health measures reducing the effect size between the intervention and control group. This, however, is consistent with the commonly observed strong placebo effect in RCTs trialling the efficacy of antidepressants for both depression^35^ and treatment resistant depression^36^. Another limitation of the study is rigorous exclusion criteria resulting in the exclusion of many potential participants. These exclusion criteria aimed to reduce the possible noise in the data from factors that could have potentially interfered with the effectiveness of the intervention. However, these strict exclusion criteria also limit the generalisability of the findings of this study and further effectiveness trials are warranted.

In conclusion, this study presents the findings from the largest randomised double blind control trial examining the effectiveness of a combination of probiotics and magnesium orotate for the treatment of MDD. Overall, the results indicate that compared with placebo, the intervention resulted in a significant short-term reduction in depression that was not maintained over an 8-weeks follow-up period. However, the significant reductions in comorbid anxiety and stress do generally appear to be maintained after the cessation of the probiotic supplement. While the results of this trial need to be replicated, they do provide evidence that a combination of *Lactobacillus acidophilus, Bifidobacterium bifidum, Streptococcus thermophilus*, Mg Orotate and CoQ10 may be a beneficial and safe treatment for MDD.

## Supplementary Information

Supplementary Information.

## Data Availability

The datasets generated during and/or analysed during the current study are not publicly available but are available from the corresponding author on reasonable request.
