# Supplementary material for: Probiotics and magnesium orotate for the treatment of major depressive disorder: a randomised double blind controlled trial
Source: Sci Rep. 2024 Sep 6;14:20841. doi: 10.1038/s41598-024-71093-z (PMC11379959; doi:10.1038/s41598-024-71093-z)
Supplement: Supplementary file 1 — Supplementary Information. [file 41598_2024_71093_MOESM1_ESM.docx]

**Supplementary Material A**

List of nutritional supplements and herbal medicines that have been shown to have antidepressant or probiotic effects:

1. SAMe (S-adenosyl methionine)
2. 5-HTP (5-hydroxytryptophan)
3. St John's Wort
4. Saffron extract
5. Magnesium
6. Kombucha
7. Kefir
8. Melatonin
9. Ashwagandha
10. Rhodiola
11. Miso
12. Chromium picolinate
13. Ginkgo biloba
14. Krill Oil
15. Fish Oil
16. Turmeric or curcumin
17. Kimchi
18. Valeriana
19. Sourdough bread
20. Sauerkraut
21. Apple Cider Vinegar
22. Xiao yao san
23. Omega 3 Oil

**Supplementary Material B**

**Life Events reported by the participants**

Negative Events:

Death of significant person in their life (N=2)

Relationship break-up (N=1)

Accident at work (N=2)

Homelessness & appearing in court (N=1)

Surgery (N=2)

Provisional dx of chronic health condition (N=1)

Positive Events:

New relationship (N=1)

Started full time work (N=1)

Change in diet (Carnivore diet) (N=1)

**Supplementary Material C**

**Adverse Events Attributed to Taking Capsules**

| **Grade** | **Attribution** | **Outcome** |
| --- | --- | --- |
| 1=Mild | 0 = Definite | 0 = Fatal |
| 2=Moderate | 1 = Probable | 1 = No recovered/resolved |
| 3=Severe | 2= Possible | 2= Recovered w/sequelae |
| 4=Life Threatening | 3 = Unlikely | 3= Recovered w/o sequelae |
| 5= Death | 4 = Unrelated | 4= Recovering/Resolving |

Only AEs rated as 0-2 in attribution were included in the table below. There were no SAEs after randomisation.

Condition A = placebo (18 AEs)

Condition B = Probiotics (17 AEs)

| **AE Category** | **Frequency Condition A** | **Frequency Condition B** | **Grade** | **Attribution** | **Outcome** |
| --- | --- | --- | --- | --- | --- |
| Abdominal pain | 2 | 2 | 1, 2 | 2 | 1, 3, 4 |
| Acne |  | 1 | 1 | 2 | 3 |
| Bloating | 3 | 4 | 1, 2 | 2 | 1, 3 |
| Constipation |  | 2 | 1 | 2 | 1, 3 |
| Diarrhoea | 1 |  | 1 | 2 | 3 |
| Emotional blunting | 1 |  | 2 | 2 | 3 |
| Fatigue | 2 |  | 1, 2 | 2 | 3, 4 |
| Flatulence | 1 | 3 | 1 | 2 | 1, 3 |
| Headaches |  | 2 | 1, 2 | 2 | 1, 3 |
| Heartburn | 1 |  | 1 | 2 | 1 |
| Insomnia (early waking) |  | 1 | 2 | 2 | 1 |
| Mental clarity impaired | 1 |  | 2 | 2 | 1 |
| Nausea | 3 |  | 1 | 2 | 1, 3 |
| Reflux | 1 | 1 | 1 | 2 | 1 |
| Restless legs |  | 1 | 1 | 2 | 3 |
| Urinary urgency | 1 |  | 1 | 2 | 1 |
| Wellbeing feelings diminished | 1 |  | 2 | 2 | 1 |
